# Large language models in real-world clinical workflows: a systematic review of applications and implementation

**DOI:** 10.3389/fdgth.2025.1659134

**Published:** 2025-09-30

**Authors:** Yaara Artsi, Vera Sorin, Benjamin S. Glicksberg, Panagiotis Korfiatis, Girish N. Nadkarni, Eyal Klang

**Affiliations:** ^1^ Azrieli Faculty of Medicine, Bar-Ilan University, Zefat, Israel; ^2^ Department of Radiology, Mayo Clinic, Rochester, MN, United States; ^3^ The Charles Bronfman Institute of Personalized Medicine, Icahn School of Medicine at Mount Sinai, New York, NY, United States; ^4^ The Windreich Department of Artificial Intelligence and Human Health, Mount Sinai Medical Center, New York, NY, United States; ^5^ The Hasso Plattner Institute for Digital Health at Mount Sinai, Icahn School of Medicine at Mount Sinai, New York, NY, United States

**Keywords:** large language models, real-world application, clinical implementation, artificial intelligence, healthcare workflows

## Abstract

**Background:**

Large language models (LLMs) offer promise for enhancing clinical care by automating documentation, supporting decision-making, and improving communication. However, their integration into real-world healthcare workflows remains limited and under characterized. This systematic review aims to evaluate the literature on real-world implementation of LLMs in clinical workflows, including their use cases, clinical settings, observed outcomes, and challenges.

**Methods:**

We searched MEDLINE, Scopus, Web of Science, and Google Scholar for studies published between January 2015 and April 2025 that assessed LLMs in real-world clinical applications. Inclusion criteria were peer-reviewed, full-text studies in English reporting empirical implementation of LLMs in clinical settings. Study quality and risk of bias were assessed using the PROBAST tool.

**Results:**

Four studies published between 2024 and 2025 met inclusion criteria. All used generative pre-trained transformers (GPTs). Reported applications included outpatient communication, mental health support, inbox message drafting, and clinical data extraction. LLM deployment was associated with improvements in operational efficiency, user satisfaction, and reduced workload. However, challenges included performance variability across data types, limitations in generalizability, regulatory delays, and lack of post-deployment monitoring.

**Conclusions:**

Early evidence suggests that LLMs can enhance clinical workflows, but real-world adoption remains constrained by systemic, technical, and regulatory barriers. To support safe and scalable use, future efforts should prioritize standardized evaluation metrics, multi-site validation, human oversight, and implementation frameworks tailored to clinical settings.

**Systematic Review Registration:**

https://www.crd.york.ac.uk/PROSPERO/recorddashboard, PROSPERO CRD420251030069.

## Introduction

The integration of large language models (LLMs) into clinical practice has sparked interest across the healthcare community (1). These technologies have the potential to enhance diagnostic accuracy, reduce administrative burden, and support clinical decision-making (2). However, while LLMs have demonstrated impressive performance in controlled retrospective settings (3, 4), their translation into clinical workflows remains inconsistent and underexplored (5).

Despite exponential growth, there remains a significant gap between developed models and real-world translation (6). The majority of explored use cases are still at the proof-of-concept stage, due to regulatory uncertainties, technical deployment barriers, privacy concerns and variable institutional readiness (7, 8).

Moreover, evaluation metrics vary widely across studies, with many reporting model performances *in silico* without assessing usability, safety, or effectiveness in real-world clinical workflow (9, 10). There is also a lack of robust post-deployment monitoring systems to better understand the impact and shifting performance of these models.

This systematic review aims to evaluate the existing literature on LLM integration into real-world clinical settings. Specifically, we assess the extent of their deployment, the clinical settings in which they are applied, the tasks they are used for, and the outcomes associated with their use. By doing so, we aim to guide future research and adoption strategies.

## Methods

### Literature search

We systematically searched the literature to identify studies describing the application of LLMs in a real-world setting. We searched MEDLINE, Google Scholar, Scopus, and the Web of Science for papers published from January 2015 to April 2025. The full search process, including Boolean operators presented here and also detailed in the Supplementary Materials.

(“large language model” OR “large language models” OR ChatGPT OR “GPT-4” OR “GPT-3” OR BERT OR “transformer model” OR “foundation model”) AND (“real-world evidence” OR “real world application” OR “clinical implementation” OR “routine practice” OR “clinical use” OR deployment OR “workflow integration”) AND (“clinical practice” OR “healthcare setting” OR hospital OR “medical setting”) AND (“original research” OR “observational study” OR “clinical study” OR “implementation study”)

In addition, we checked the reference lists of selected publications and the “Similar Articles” feature in PubMed, to identify additional publications. Ethical approval was not required, as this is a systematic review of previously published research and does not include individual participant information. Our study followed the Preferred Reporting Items for Systematic Reviews and meta-analyses (PRISMA) guidelines (11). The study is registered with PROSPERO (CRD420251030069).

### Study selection

We included studies conducted in real-world clinical care settings, such as hospitals, clinics, ambulatory, inpatient, outpatient, emergency, and primary care involving clinicians and/or patients. The intervention was an LLM-enabled tool integrated into live workflows. Eligible comparators included usual care pre–post designs. Outcomes encompassed workflow, efficiency, usability, adoption, clinical impact, and safety vs. risk. We excluded simulation-only studies (including vignette-based evaluations not used to guide real patient care), bench evaluations without deployment, and non-LLM NLP. All search results were imported into a single CSV table and deduplicated. Two authors (YA and VS) independently screened titles and abstracts for relevance. Potentially eligible articles were retrieved in full text and assessed by YA and VS. Discrepancies were resolved by a third author (EK).

### Inclusion and exclusion criteria

**Figure 1:**
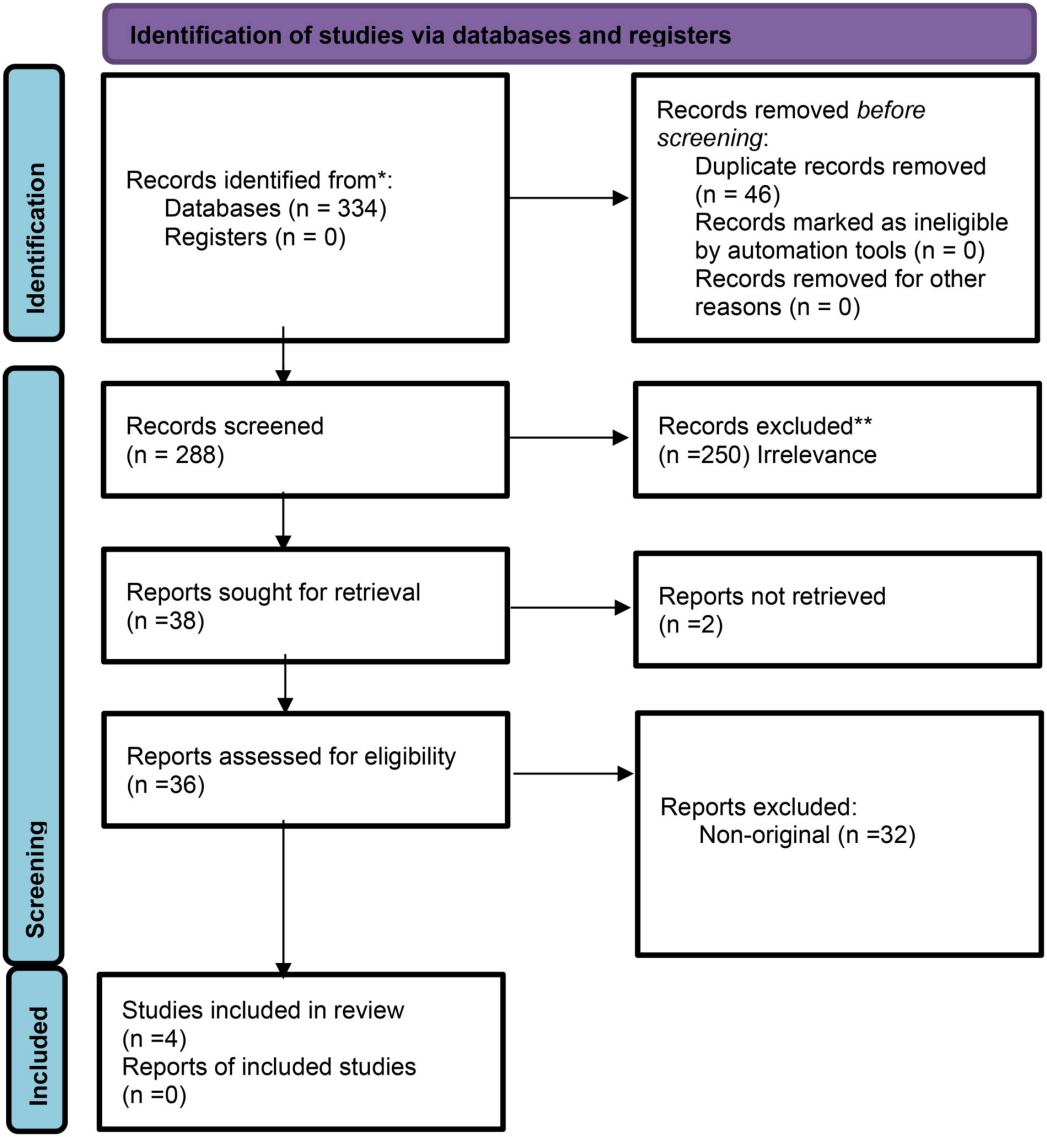
Flow diagram of inclusion and exclusion process.

Full-text peer-reviewed publications in English focusing on LLMs integration and deployment in real-world clinical workflow were included. We excluded non-English articles, non-original research, non-peer-reviewed publications, studies that did not assess LLMs, and studies that did not explicitly assess LLMs in real-world settings. Figure 1 presents the flow diagram of the screening and inclusion process.

### Quality assessment

**Figure 2:**
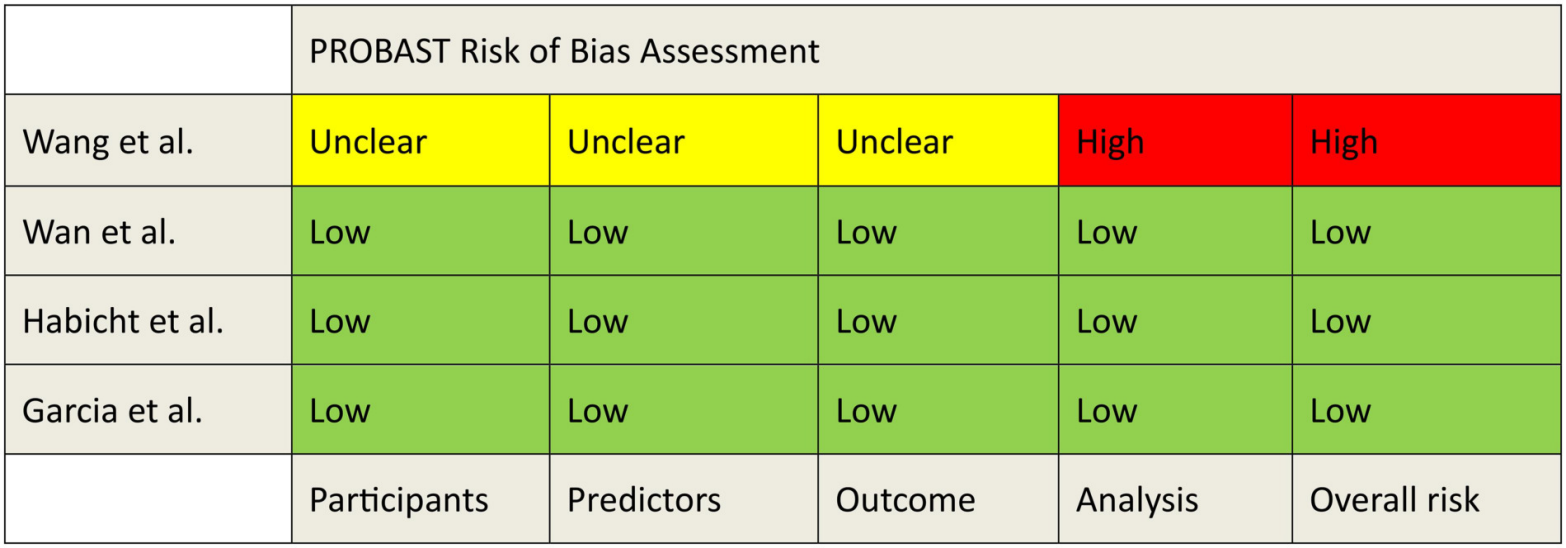
PROBAST risk of bias assessment.

**Figure 3:**
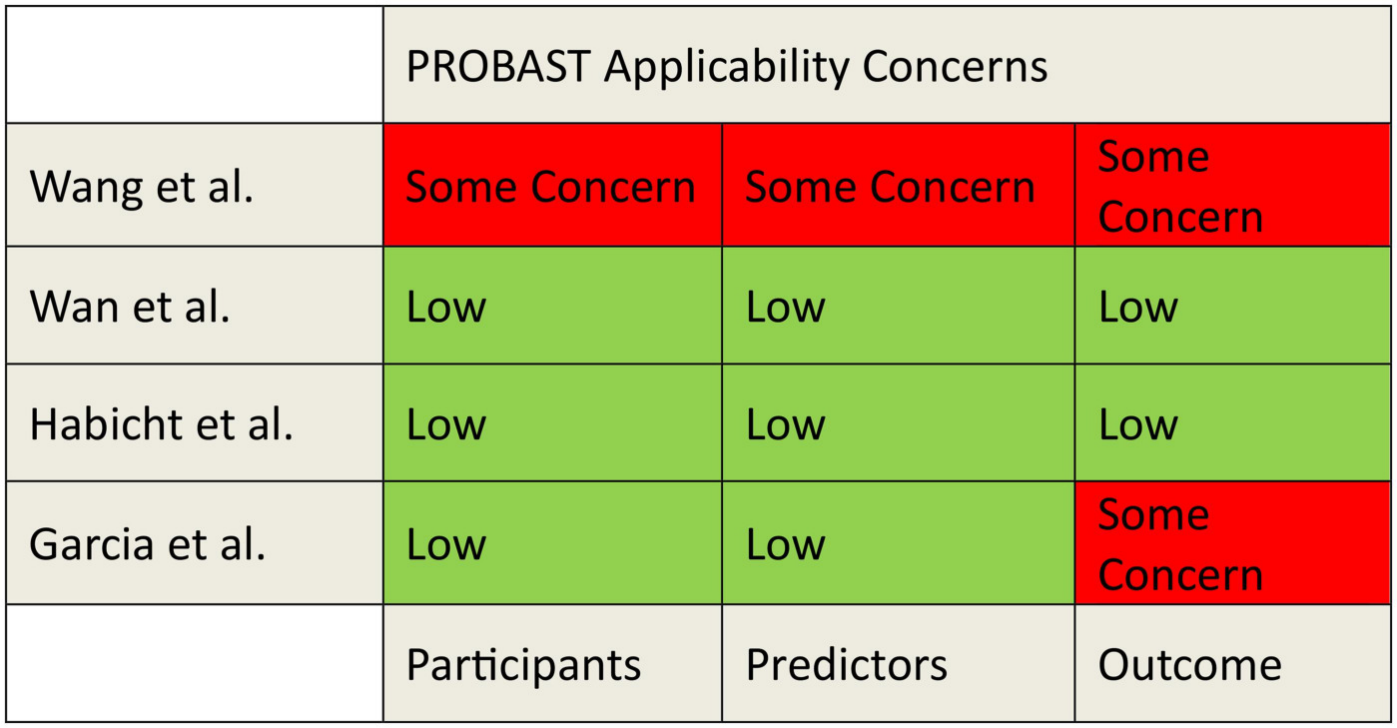
PROBAST assessment for applicability.

The risk of bias and applicability was evaluated using the PROBAST tool (Figures 2, 3). A detailed assessment of the studies using the PROBAST tool is detailed in the Supplementary Material.

### Fundamental concepts

An overview of fundamental concepts in AI is included in the Supplementary Material, along with visual hierarchy shown in Supplementary Figure S1.

## Results

### Study selection and characteristics

Four studies were included in this review, published between March 2024 and March 2025. All studies utilized generative pre-trained transformers (GPT) (100%). Two studies focused on patient services, including LLM-supported communication during outpatient intake and response to patient messages (50%). Two studies' focus areas were on data extraction (50%), one applied LLM as a support tool (25%) (Table 1).

**Table 1:** General features of reviewed studies.

Study	Setting	Population	Intervention (LLM task)	Comparator/design	LLM specifics	Sample size	Type of data
Wang et al. (12)	Chinese hospital	Not reported	Data extraction	Not reported	ChatGLM2-6B	Not reported	Quantitative
					LLaMA2-7B		
					Fine-tuning (3 rounds) with few-shot + RAG		
Wan et al. (13)	Outpatient reception workflows (2 medical centers)	Not reported	Patient intake and reception	Not reported	GPT-3.5-Turbo	2,164 patients	Quantitative
					Fine-tuned with site-specific knowledge (SSPEC) + prompt template		
					GPT-4 evaluator + RAG		
Habicht et al. (14)	Group-based cognitive behavioral therapy (UK talking therapies)	Not reported	Therapy support system	Not reported	GPT-4	244 patients	Quantitative
					No model fine-tuning reported		
Garcia et al. (15)	Primary care, gastroenterology, hepatology	Not reported	Clinical communication support	Not reported	GPT-3.5 Turbo GPT-4	197 clinicians (162 analyzed)	Quantitative
					No domain-specific training		

**Figure 4:**
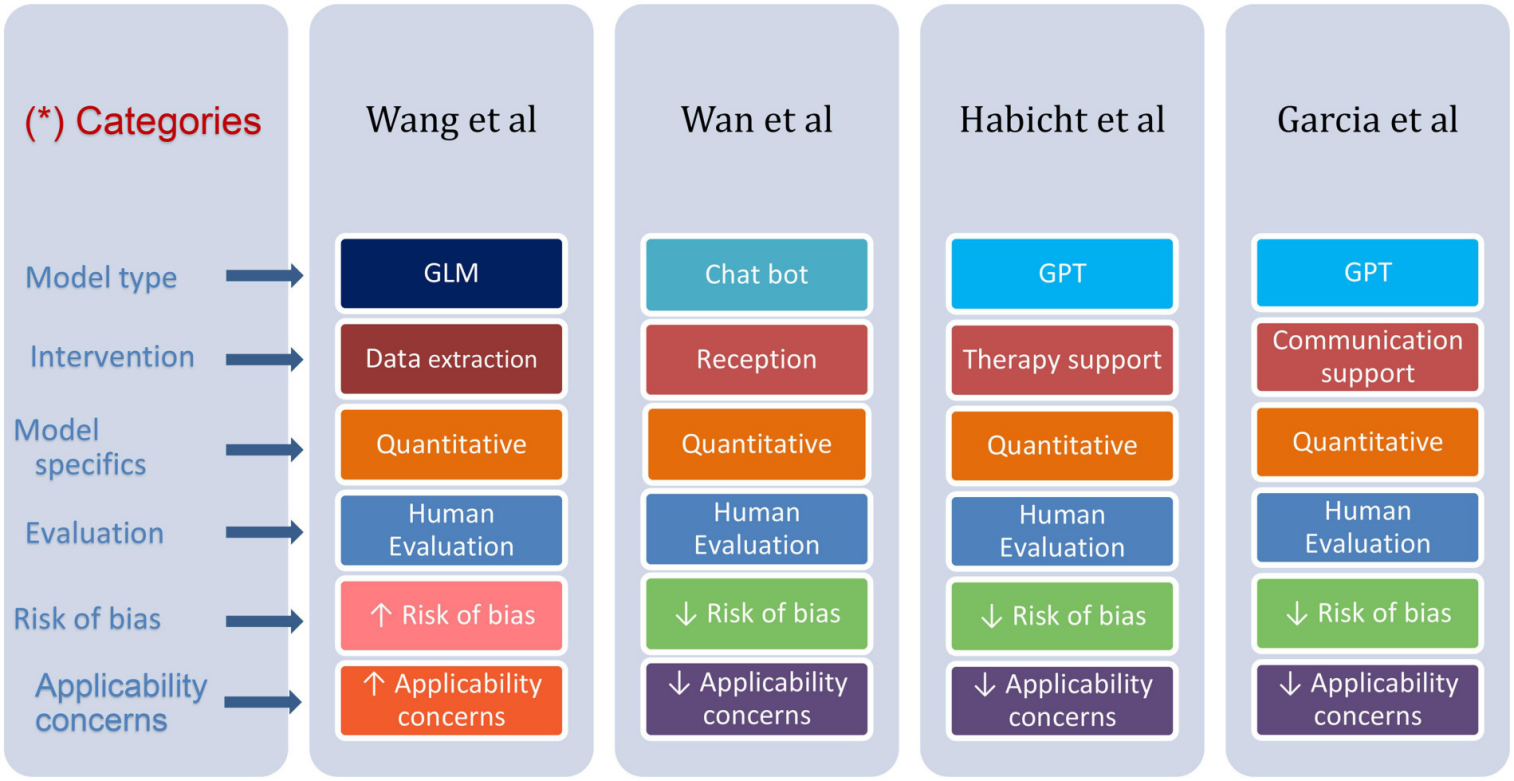
Differences and similarities in various parameters presented in the reviewed studies. (*) Each row has its distinctive color, where color tone change emphasizes a difference.

The results of the studies are summarized in Table 2. Figure 4 provides an overview of the characteristics of the included studies.

**Table 2:** Evaluation metrics and key results of reviewed studies.

Study	Evaluators	Evaluation metrics	Evaluation tools	Key results
Wang et al. (12)	Human	Data transcription time reduction	Manual annotation	80.7% reduction
		Accuracy of data extraction	Time logging	77.13% for free-text
				98.72% for structured medication data
				For LLaMA2-7B lower accuracy, especially for vital signs and family history extraction
Wan et al. (13)	Human (patients & clinical staff)	Patients’ satisfaction	Likert-scale surveys	Higher satisfaction (3.91 vs. 3.39, *P* < 0.001)
		Emotional response & response quality	Structured questionnaires	Reduced repeated questions (3.2% vs. 14.4%)
				Lower negative emotions (2.4% vs. 7.8%)
				Improved integrity, empathy, and readability of responses
Habicht et al. (14)	Human (therapists & participants)	Session attendance & dropout rate	Clinical records	23 percentage point reduction in dropout
		Reliable improvement	Qualitative feedback surveys	Higher rates of reliable improvement recovery
		Recovery rates	Standardized depression (PHQ-9) & Anxiety (GAD-7) questionnaires	Strong dose-response relationship between app engagement and clinical outcomes
Garcia et al. (15)	Human (clinicians)	Inbox utilization rate	System usage logs	Mean draft utilization: 20%
				75% of messages had AI drafts
		Physician task load score	Surveys (pre- and post-intervention)	Significant reduction in task load (−13.87 points)
		Work exhaustion score		Significant reduction in work exhaustion (−0.33 points)
		User satisfaction	Net promoter score (NPS)	Favorable: primary care physicians/APPs (13), primary care clinical pharmacists (71), GI/hepatology nurses (50)
				Unfavorable: primary care nurses (−60), GI/hepatology physicians/APPs (−19)

### Descriptive summary of results

Wang et al. (12) evaluated ChatGLM2-6B for real-world data extraction, and did not report a patient count. The model achieved an 80.7% reduction in transcription time. The accuracy varied by data type, 77.13% for free text and 98.72% for structured medication data. In comparison, the LLaMA2-7B model showed lower accuracy, especially for vital signs and family history (Table 2).

Wan et al. (13) randomized 2,164 outpatients across two medical centers to nurse-only vs. nurse–SSPEC workflows. The nurse–SSPEC model improved patient satisfaction, reduced repeated questions (3.2% vs. 14.4%), and lowered negative patient emotions (2.4% vs. 7.8%). It also enhanced response quality in integrity, empathy, and readability (Table 2).

Habicht et al. (14) assessed a GPT-4–powered AI tool in group-based CBT (*n* = 244 patients) and found it improved clinical outcomes. The AI group had more session attendance, fewer missed appointments, and a 23-percentage point lower dropout rate than those using standard worksheets—higher engagement correlated with better adherence and outcomes. Qualitative feedback also noted improved self-awareness, mindfulness, and practical use of CBT techniques (Table 2).

Garcia et al. (15) evaluated GPT-3.5 Turbo and GPT-4 for generating draft replies to patient messages in gastroenterology, hepatology, and primary care. The AI drafts improved efficiency and reduced clinician workload without compromising communication quality. Enrolled 197 clinicians, of whom 162 were included in the final analysis; draft utilization averaged 20%, with 75% of messages receiving AI-generated replies. While time spent on inbox tasks did not significantly change, clinicians reported reduced task load and work exhaustion. User feedback raised concerns about message tone, length, and relevance (Table 2).

Limitations and challenges discussed or inferred in the reviewed studies are presented in Table 3.

**Table 3:** Limitations and challenges in reviewed studies.

Study	Challenges	Domain impact	Observed or inferred
Wang et al. (12)	Moderate accuracy variability (77.13% for free-text extraction) Lower performance compared to LLaMA2-7B in some fields	Data extraction accuracy	Observed
Wan et al. (13)	Need for careful prompt design Reliance on nurse oversight	Patient communication Workflow efficiency Scalability and generalizability	Inferred
Habicht et al. (14)	App retention decline (only 19.3% engaged by week 6)	Mental health outcomes Patient engagement Tool sustainability	Observed
Garcia et al. (15)	Variability in tone, message relevance, and adoption Modest impact on inbox time Concerns about over-reliance on AI	Administrative burden Clinician well-being	Observed

## Discussion

**Figure 5:**
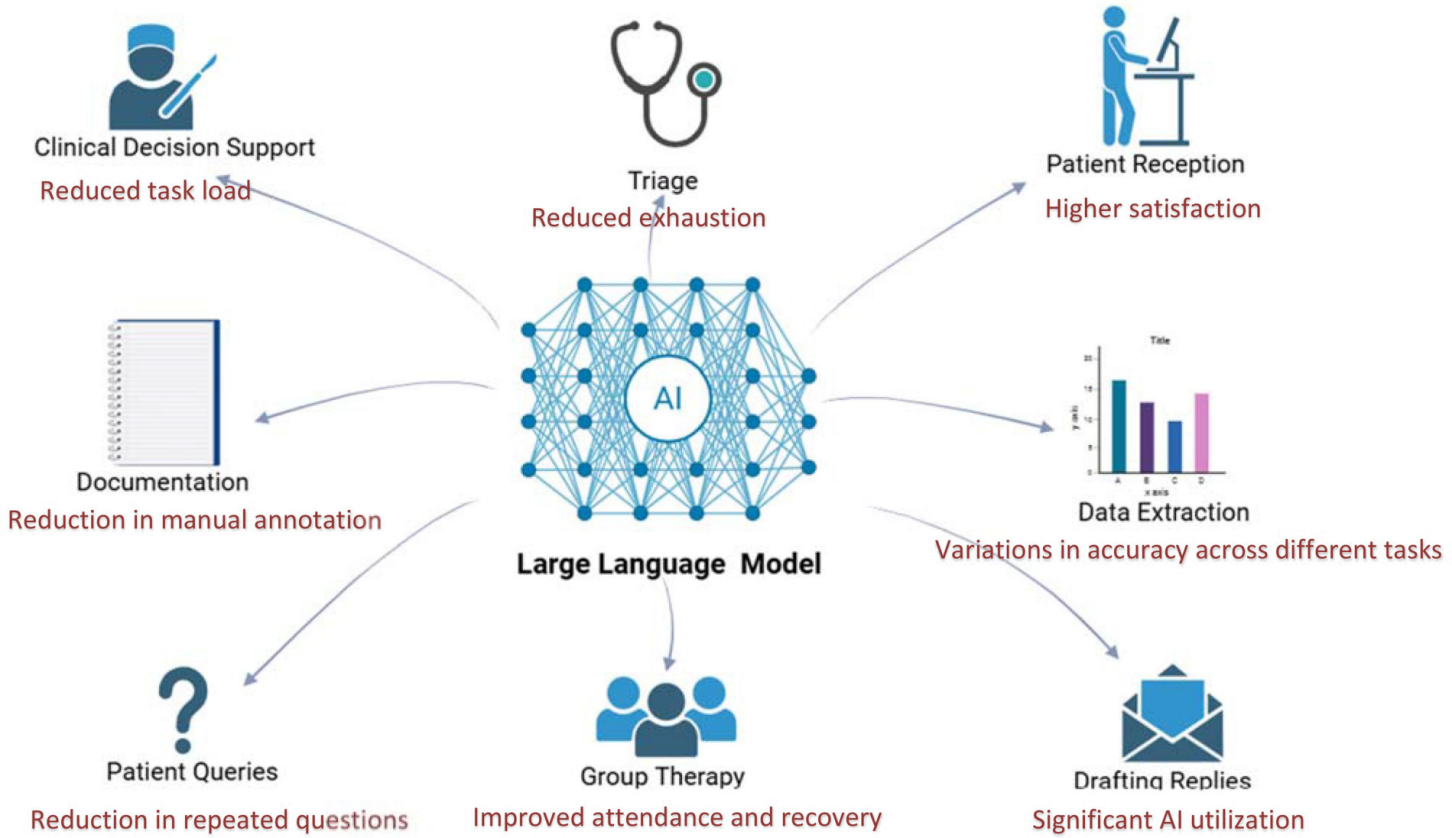
LLM application in various fields in real-world setting, integrated with summarized results from reviewed studies. Created using Biorender, licensed under Academic License.

LLMs show promise in real-world clinical workflows (16), with the potential to enhance many fields in clinical care. We illustrate this in Figure 5, showcasing various clinical domains with key results from the reviewed studies (Figure 5). However, their implementation remains early-stage and context-dependent. Our synthesis across four deployed implementations indicates consistent benefits in task burden and clinician experience, alongside improvements in selected patient-facing outcomes. At the same time, effects remain context-dependent, varying by role, setting, task type, and integration depth. In this context, our synthesis indicates that LLMs function less as universal accelerators and more as context-sensitive amplifiers of specific tasks, with the clearest benefits emerging when models are embedded in existing tools and supervised. Several studies demonstrated clear empirical benefits. For example, Habicht et al. (14) showed that GPT-4 as a therapy support tool reduced therapy dropout rates and improved clinical outcomes in group interventions. Wan et al. (13) reported that the site-specific LLM chatbot (SSPEC) reduced repeated interactions and negative patient emotions. Garcia et al. (15) found that GPT-generated draft replies decreased clinician task load and work exhaustion across several clinical settings. These findings suggest that the value of LLMs is realized not simply by model capability but by deliberate product-workflow fit, a bounded task, appropriate timing in the clinical journey, and clear human supervision and handoff.

Across included studies, three implementation patterns consistently enabled success. Retrieval-augmented generation (RAG) with prompt templates to ground outputs in local knowledge, guardrails, and escalation tiers (including evaluator models and human handoffs) to manage safety and appropriateness, and EHR integration to address privacy and reduce workflow friction. Conversely, role-specific dissatisfaction, and retention decay in patient-facing flows emerged as failure modes when these patterns were absent or inconsistently applied.

Despite encouraging signals, translation and scale remain constrained. In Wang et al. (12), while ChatGLM2-6B achieved a reduction in transcription time, accuracy varied significantly across data types. LLaMA2-7B performed notably worse in comparison. These discrepancies accentuate the importance of task specificity and local calibration in AI deployment.

While LLMs can improve operational efficiency and clinical outcomes, widespread adoption is limited by systemic barriers and arbitrary evaluation metrics (17). These include heterogeneous EHR systems and unclear standards for performance evaluation. Future research should focus on efficient prospective validation and developing clear standardized evaluation metrics. We propose a list of possible standardized evaluation domains and metrics detailed in Table 4.

**Table 4:** Proposed standardized evaluation domains and metrics for real-world LLM implementations.

Domain	Recommended metric
Model performance & calibration	AUROC/AUPRC
	Accuracy
	Precision
	Recall
	F1-score
	Calibration (Brier score, ECE)
	Coverage/abstention rate for selective deferral
Clinical impact	Patient outcomes
	Condition-specific outcomes or PROs
	Diagnostic/triage concordance
	Time to treatment or appropriate referral
	Guideline-adherence delta
	Tests/visits avoided
Workflow efficiency	Task time per case
	Response/turnaround time
	Time-to-decision.
Usability & adoption	SUS or UMUX-Lite
	Perceived usefulness & ease-of-use (TAM)
	NASA-TLX (task load)
	% AI-assisted tasks
	Clinician reported task load
Reliability & monitoring	Rate of false negatives/positives
	Override rates
	Failure rate
	Latency
	Performance drift over time
	Post-deployment incident reports
	Rollback frequency
Deployment fidelity	User adherence to intended use rates
	Percentage of AI-assisted tasks
	Prompt/template adherence
	Version tracking
Generalizability	Cross-site performance variance
	Calibration curves
Safety & risk	Hallucination rate (overall and clinically significant)
	Harmful/unsafe recommendation rate
	Override rate (and appropriateness)
	Near-miss and adverse event counts
	Severity-weighted error index
	PHI leakage or privacy breach rate
	Alert-fatigue index

Translating LLMs from experimental settings into clinical practice remains a challenge. Clinical implementation is frequently delayed by regulatory barriers, including classification as software-as-a-medical-device (SaMD), which necessitates lengthy regulatory approval processes and extensive local validation (18). These processes contribute to version lag, whereby newer models become available before prior versions are deployed or evaluated. Furthermore, performance may degrade over time due to evolving clinical documentation, user behavior, or patient populations (8). These challenges underscore the need for post-deployment monitoring frameworks and standardized scientific reporting to ensure model safety, performance, and generalizability in clinical environments (17, 19).

### Strategies for the future

Several strategic approaches should be considered to facilitate the broader adoption of LLM technologies in clinical workflows. Local adaptation of models is essential, as performance can vary significantly depending on institutional characteristics such as data quality, documentation styles, and patient demographics. Ensuring that models are trained and validated on local data can improve generalizability and clinical relevance. Also, incorporating a human expert oversight framework can improve safety, accountability, and user trust.

This review highlights the need for comparable outcome definitions. Studies frequently mix denominators, time windows, and units, hindering synthesis. Outcomes are also unevenly distributed. Workflow or experience measures are common, while downstream clinical outcomes and severity-weighted safety indicators are rare. To improve comparability, a standardized evaluation set with consistent units, denominators, and time windows are required, with selective deferral and severity-aware incident metrics. Adopting shared reporting conventions would make future evidence more cumulative, enable meta-analytic techniques where appropriate, and clarify trade-offs between efficiency, quality, and risk across sites.

AI tools should be designed with task specificity; models tailored to distinct clinical functions, such as triage, documentation, or medication extraction, are more likely to achieve meaningful utility. This can be achieved using fine-tuning (20) or Retriever-Augmented Generation (RAG) (21).

A standardized set of metrics should be developed to support consistency and facilitate future evaluations. We propose our set of metrics in Table 4. Also, involving clinicians and end users in the development and implementation process ensures that tools are aligned with real-world workflow needs, increasing user acceptance. Collecting user feedback during deployment can guide iterative model refinement and usability improvements.

Safety mechanisms and override options are crucial to prevent unintended consequences such as clinical errors, automation over-reliance, data bias, and alert fatigue (22). These risks can disrupt workflows and raise ethical concerns about accountability. Human oversight and ongoing monitoring are essential to ensure AI supports, rather than compromises, clinical care (19).

Ensuring that AI tools are clinically effective requires more than technical performance. This includes interoperability with EHRs, standardized evaluation metrics, and human-centered design features like transparency and clinician oversight. By applying these strategies, AI can move beyond experimental use to become a trusted part of routine clinical care.

This review has several limitations. First, the number of eligible studies evaluating LLMs in real-world clinical workflows remains limited, reflecting the early stage of implementation research in this domain. Our search emphasized real-world clinical settings, which may have excluded studies that did not explicitly use setting descriptors. Nonetheless, multi-database coverage and related-article screening reduce the likelihood of missing eligible deployments. Second, the heterogeneity in study design, evaluation methods, clinical settings, and outcome reporting precluded formal meta-analysis. Third, most included studies were conducted in high-resource settings, potentially limiting the generalizability of findings to low- and middle-income countries. Additionally, some of the studies relied on self-reported outcomes or lacked long-term follow-up, which may introduce reporting bias or fail to capture sustained clinical impact. Finally, despite efforts to capture a comprehensive set of studies, some relevant work may have been missed due to language restrictions, database coverage, or publication lag.

## Conclusion

LLMs demonstrate early but uneven success in real-world integration, with empirical improvements in efficiency and user satisfaction. They demonstrate encouraging but context-dependent benefits in real-world clinical workflows, and their effects varied by role, task, and site, underscoring that outcomes depend as much on implementation choices as on the underlying model family. However, challenges related to generalizability, interoperability, and evaluation must be addressed to ensure scalable and safe adoption. LLMs' outcomes are situation-specific and site-specific, underscoring the need for multi-site validation, transparent version reporting, and post-deployment monitoring for safety and equity. For health systems, the immediate implication is to prioritize implementation architecture, such as RAG pipelines, EHR-proximal integration, and measure outcomes with standardized units and denominators. Future research should prioritize prospective multi-center validation, using standardized metrics, and end-user collaboration with evaluation of role-specific impacts and comparative studies of design patterns to support the responsible and effective use of AI in clinical care.

## Data Availability

The original contributions presented in the study are included in the article/Supplementary Material, further inquiries can be directed to the corresponding author.
